# Pathological Roles of Wild-Type Cu, Zn-Superoxide Dismutase in Amyotrophic Lateral Sclerosis

**DOI:** 10.1155/2012/323261

**Published:** 2012-07-01

**Authors:** Yoshiaki Furukawa

**Affiliations:** Laboratory for Mechanistic Chemistry of Biomolecules, Department of Chemistry, Keio University, 3-14-1 Hiyoshi, Kohoku-ku, Yokohama, Kanagawa 223-8522, Japan

## Abstract

Dominant mutations in a Cu, Zn-superoxide dismutase (SOD1) gene cause a familial form of amyotrophic lateral sclerosis (ALS). While it remains controversial how SOD1 mutations lead to onset and progression of the disease, many *in vitro* and *in vivo* studies have supported a gain-of-toxicity mechanism where pathogenic mutations contribute to destabilizing a native structure of SOD1 and thus facilitate misfolding and aggregation. Indeed, abnormal accumulation of SOD1-positive inclusions in spinal motor neurons is a pathological hallmark in SOD1-related familial ALS. Furthermore, similarities in clinical phenotypes and neuropathology of ALS cases with and without mutations in *sod1* gene have implied a disease mechanism involving SOD1 common to all ALS cases. Although pathogenic roles of wild-type SOD1 in sporadic ALS remain controversial, recent developments of novel SOD1 antibodies have made it possible to characterize wild-type SOD1 under pathological conditions of ALS. Here, I have briefly reviewed recent progress on biochemical and immunohistochemical characterization of wild-type SOD1 in sporadic ALS cases and discussed possible involvement of wild-type SOD1 in a pathomechanism of ALS.

## 1. Introduction

Amyotrophic lateral sclerosis (ALS) is a fatal paralytic disorder caused by degeneration of motor neurons in the brain and spinal cord. One tenth of the total ALS cases occur in familial forms (fALS), while the other is sporadic with no known genetic components (sALS) [1]. The clinical phenotypes between fALS and sALS are usually indistinguishable; therefore, the understanding of the genetic cause in fALS will be also relevant in sALS cases. In spite of tremendous numbers of clinical, pathological, and biochemical studies, however, we still have no cure for this devastating disease.

In 1993, there was a milestone event in the studies on ALS; mutations in *sod1* gene were identified to be associated with fALS [2, 3]. Together with the recent findings on the pathogenic mutations in *c9orf72* gene [4, 5], the mutations in *sod1* are now prevailed in fALS (~20% of total familial cases), and more than a hundred mutations in *sod1* have been found to be associated with fALS (ALSoD: http://alsod.iop.kcl.ac.uk/). The *sod1* gene encodes a protein, Cu, Zn-superoxide dismutase (SOD1), which catalyzes the detoxification of superoxide anion by dismutation into oxygen and hydrogen peroxide [6]. Retardation of such SOD1 activity with mutations had been initially expected to be pathogenic in fALS; however, knockout of *sod1* gene in mice did not reproduce the ALS-like symptoms [7], and also, some of the fALS-causing mutations did not alter the dismutation activity of SOD1 [8]. Instead, the severe progressive neurodegeneration can be well reproduced in transgenic mice expressing human SOD1 with ALS-causing mutations [9]. These observations support that SOD1 gains some toxic properties by pathogenic mutations.

 How mutations in *sod1* gene exert toxicity eventually causing the death of motor neurons remains obscure, but it has been widely accepted that an SOD1 protein increases its propensity for insoluble aggregation by pathogenic mutations [10]. Indeed, the surviving motor neurons in spinal cords of SOD1-related fALS cases are characterized by the presence of Lewy body-like hyaline inclusions (LBHI) and skein-like inclusions [11] that are composed of misfolded forms of mutant SOD1 proteins [12]. Notably, furthermore, several antibodies specifically recognizing misfolded SOD1 have detected conformational abnormalities of wild-type SOD1 in a subset of sALS cases (*vide infra*). Understanding pathological or even pathogenic roles of wild-type SOD1 in ALS may contribute to the etiology of sALS, which occupies almost 90% of total ALS cases. Regarding roles of SOD1 mutations in the fALS pathomechanism, tremendous numbers of *in vitro* and *in vivo* studies have been performed, and many excellent reviews have been published so far (e.g., [9, 13–17]). Accordingly, in this paper article, I have summarized recent development on our understanding of roles of wild-type SOD1 in ALS.

## 2. Inherent Propensities of Wild-Type SOD1 for Aggregation

 Structural stability as well as enzymatic activity of SOD1 is controlled by the post-translational processes that include the binding of copper and zinc ions and the formation of a highly conserved intramolecular disulfide bond [18, 19]. Copper ion bound in SOD1 serves as an active site for dismutation of superoxide anion, and the structural stability of SOD1 is significantly increased upon binding of a zinc ion and the formation of a disulfide bond [18]. Indeed, holo-SOD1 with a disulfide bond has been known as one of the most stable proteins with around 90°C of the melting temperature (*T*_*m*_) [20], which decreases to as low as 43°C upon the disulfide reduction and demetallation in SOD1 [18]. Retardation of the post-translational processes, therefore, increases a chance for SOD1 to be misfolded and aggregated. In normal healthy conditions, wild-type SOD1 is correctly processed to acquire metal ions and a disulfide bond, which prevents SOD1 from aggregation [19]. Importantly, however, it is notable that wild-type SOD1 has been shown *in vitro* to form amyloid-like fibrillar aggregates in the disulfide-reduced and apo state [21, 22]. This result implies that wild-type SOD1 is also susceptible to aggregation and becomes pathogenic, when the post-translational processes are disrupted.

## 3. Immunodetection of Wild-Type SOD1 with Aberrant Conformations in sALS Cases

 Recently, mutations in several genes other than *sod1* have been identified in a subset of fALS cases, which include genes encoding TDP-43 [23–26], FUS [27, 28], and optineurin [29]. These proteins with pathogenic mutations constitute pathological inclusions, but it is also important to note that immunoreactivities to TDP-43, FUS, and optineurin have been detected in inclusions in sALS cases even without any mutations in those proteins [30–33]. Although it remains unclear what triggers the aggregation of those wild-type proteins in sALS cases, these observations have implied the presence of a common pathomechanism converging familial and sporadic forms of ALS.

In contrast, wild-type SOD1 appears not to be involved in the formation of inclusions in sALS cases (*vide infra*), although fALS cases with mutations in *sod1* gene invariably exhibit SOD1-immunoreactive inclusions. Furthermore, these SOD1-positive inclusions in SOD1-fALS cases are not immunostained with antibodies to TDP-43 [32, 33], FUS [31], and optineurin [30], while some SOD1-fALS cases exhibit two types of mutually exclusive inclusions: SOD1-positive and TDP-43-positive inclusions [34]. Distinct pathomechanisms would, therefore, exist between SOD1-fALS and the other ALS cases. Despite this, it is important to consider that, even without mutations, wild-type SOD1 exhibits pathological changes in a subset of sALS cases. Indeed, formation of TDP-43/FUS-positive inclusions in sALS as well as fALS with *fus* mutations has been recently reported to be associated with misfolding of wild-type SOD1 proteins [35]. In addition, chronic overexpression of wild-type human SOD1 in mice causes several neurodegenerative changes including mitochondrial vacuolization and loss of spinal motor neurons [36], implying that SOD1 can be pathogenic even without fALS-causing mutations.

Involvement of wild-type SOD1 in the pathological inclusions has been first pointed out by Shibata et al. [37, 38]. They have found LBHIs in 10 out of 20 sALS cases, and some of those inclusions in anterior horn cells of spinal cord have shown intense immunoreactivity toward the rabbit antisera raised against human SOD1 [39]. Interestingly, the proportion of LBHIs with SOD1 immunoreactivity varied from case to case (7–60%), which is in sharp contrast to the fALS cases with mutations in *sod1* gene (100%). They have, however, not verified whether their sALS cases have mutations in *sod1* gene. Because mutations in *sod1* gene have been found in less than 5% of total sALS cases [40], care will be needed to interpret the SOD1-immunoreactive inclusions in sALS cases.

Watanabe et al. have also observed hyaline inclusions in 6 out of 17 sALS cases, but those inclusions were not immunoreactive to a rabbit polyclonal antibody raised against a Asp^124^–Lys^136^ peptide of human SOD1 [41]. No description was again found in their paper on whether the sALS cases had mutations in *sod1* gene. Despite this, it is interesting to note that skein-like inclusions were immunoreactive to a protein, CCS, in 1 out of 17 sALS cases. CCS is a copper chaperone for SOD1, which specifically delivers a copper ion [42] and also introduces a conserved disulfide bond in SOD1 [19]. Recruitment of CCS into insoluble inclusions may thus increase the intracellular fraction of inactive SOD1 and facilitate the formation of abnormal SOD1 with pathological conformations. Nonetheless, CCS immunoreactivity was not observed in the hyaline inclusions in SOD1-related fALS cases with A4V mutation [41], and knockout of *ccs* gene in a mouse did not produce any pathological changes as well as ALS-like symptoms [43]. In contrast to the previous report by Shibata et al. [37, 38], therefore, these observations have supported less contribution of wild-type SOD1 and CCS to the pathologies in sALS cases.

Native SOD1 has been well known to exist as a tight dimer, and the monomerization is often considered to be involved in the misfolding/aggregation pathway of SOD1 [44]. To detect misfolded SOD1 in affected tissues of ALS, it is quite reasonable to make an antibody that specifically recognizes monomeric SOD1. For that purpose, the SEDI (SOD1-exposed-dimer-interface) antibody was prepared by rabbit immunization with a Arg^143^–Ile^151^ peptide at the dimer interface of SOD1, which is buried and inaccessible in the dimeric state but becomes exposed upon monomerization [45]. Indeed, this SEDI antibody has successfully immunostained hyaline inclusions in motor neurons of the fALS cases with *sod1* mutation (A4V, A4T, V14M, ΔG27/P28, I113T); however, inclusions were not detected with the SEDI antibody in all of 13 sALS cases and 1 non-SOD1 fALS case, where no mutations in *sod1* gene were confirmed [46].

Another antibody (USOD) has also been produced by rabbit immunization with an SOD1 fragment from Leu^42^ to His^48^, which constitutes an internal hydrophobic core of the native structure [47]. This region (Leu^42^–His^48^) is supposed to become exposed only when SOD1 is extensively misfolded; therefore, the USOD antibody specifically binds extensively misfolded SOD1 but not native dimeric as well as folded monomeric states. Similar to the SEDI antibody, the USOD antibody has detected pathological inclusions in fALS cases with *sod1* mutations (A4V, ΔG27/P28) but not in sALS cases without *sod1* mutations [47]. Despite this, these results would not be sufficient to conclude less contribution of wild-type SOD1 to the disease pathologies, because wild-type SOD1 in sALS cases may adopt abnormal conformations that cannot be recognized with the SOD1 antibodies above.

Indeed, Forsberg et al. [48] have systematically prepared a series of rabbit polyclonal antibodies that are raised against the SOD1 fragments corresponding to Ala^4^–Phe^20^, Glu^24^–Val^39^, His^43^–Cys^57^, Cys^57^–Gly^72^, His^80^–Asp^96^, Glu^100^–Arg^115^, and Asn^131^–Gln^153^, among which Ala^4^–Phe^20^, Cys^57^–Gly^72^, and Asn^131^–Gln^153^ antibodies, in particular, reacted strongly with chemically denatured SOD1 but hardly at all with native SOD1. Importantly, these antibodies detected small round inclusions in spinal motor neurons of all the 29 sporadic and 8 familial ALS cases lacking mutations in *sod1* gene; the abundance of such inclusions was much smaller in controls without neurological diseases. Using Cys^57^–Gly^72^ and Asn^131^–Gln^153^ antibodies, furthermore, misfolded wild-type SOD1 in the form of granular aggregates was regularly detected in the nuclei of astrocytes, microglia, and oligodendrocytes in ALS patients lacking *sod1* mutations [49]. These small granular inclusions in sALS cases are distinct from the inclusions in fALS with *sod1* mutations, which are often characterized with large Lewy body-like morphologies [11]. In sALS cases, therefore, wild-type SOD1 would not participate in the formation of Lewy body-like inclusions, but it is certain that wild-type SOD1 undergoes some conformational changes under pathological conditions.

There has been also an interesting antibody called C4F6 for detection of abnormal SOD1 conformations, which is a mouse monoclonal antibody raised against recombinant apo-SOD1 with G93A mutation [50]. In *in vitro* experiments, an epitope of C4F6 was found to include specific conformations realized in SOD1^G93A^ but not wild-type SOD1. Interestingly, however, the structure of wild-type SOD1 was changed by treatment with H_2_O_2_ so that it became detectable with C4F6 [51]. H_2_O_2_ has been found to primarily oxidize the free thiol group at Cys^111^ into sulfonic acid, which also appears to occur even under physiological conditions [52]. Furthermore, such C4F6-reactive conformers of oxidized SOD1 inhibits fast axonal transport to a degree reminiscent of that of mutant SOD1 [51]; therefore, Cys^111^-oxidized SOD1 will share important structural and toxic features with fALS-causing mutant SOD1 proteins.

As expected, skein-like inclusions in spinal motor neurons of SOD1-related fALS cases (SOD1^A4V^) were immunoreactive with C4F6 antibody [53]. Notably, positive C4F6 staining was also observed in 4 out of 9 sALS cases, while no large skein-like and Lewy body-like inclusions immunoreactive with C4F6 were reported in sALS cases [51]. Rather, the diffused staining patterns of C4F6 antibody in sALS cases suggested that the pathological forms of wild-type SOD1 remain relatively soluble. Indeed, biochemical analysis did not support elevated levels of insoluble wild-type SOD1 in sALS cases [51]. Recently, the diffused staining with C4F6 was reported to be seen not only in ALS cases, but also in motor neurons from patients dying with other neurological diseases and in non-neurological controls [53]. Replacement of the C4F6 antibody with normal mouse serum also resulted in diffused staining in some motor neurons of sALS cases, implying the significant contributions of nonspecific background to the diffused staining with C4F6 [53]. It thus requires further investigation to clarify whether C4F6 antibody detects misfolded wild-type SOD1 in sALS cases without *sod1* mutations.

## 4. Biochemical Characterization of Wild-Type SOD1 in sALS Cases

 A very few studies have been performed to characterize molecular signatures of wild-type SOD1 purified from sALS cases, but several biochemical approaches have been attempted to illuminate pathological conformers of wild-type SOD1 in non-SOD1 sALS cases. One of such approaches is based upon the idea that different protein conformers will exhibit distinct accessibility toward chemical compounds. Given that some of covalent modifications are well expected to magnify conformational differences of a protein molecule, Gruzman et al. [54] have first prepared SOD1 from sALS tissue samples and then covalently modified accessible lysine residues in SOD1 with *N*-hydroxysulfosuccinimide-long chain-biotin (Sulfo-NHS-LC-Biotin). After biotinylation, SOD1 species with electrophoretic mobility corresponding to 32 kDa of molecular weight became evident in sALS as well as fALS cases. Such biotinylation-dependent 32-kDa species of SOD1 also existed in some of normal healthy controls and patients with other neurological diseases, which was, however, reported to be statistically insignificant. Accordingly, a small fraction of wild-type as well as mutant SOD1s would adopt such abnormal conformations that are common to all ALS cases, but it still remains elusive how SOD1 changes its conformation under pathological conditions.

As mentioned above, antibodies raised against Glu^24^–Val^39^ and Cys^57^–Gly^72^ of SOD1 exhibit high immunoreactivity specifically to abnormal misfolded but not natively folded SOD1 [48]. By using ELISA with those antibodies, Zetterström et al. have attempted to quantify amounts of pathological forms of SOD1 in ALS cases [55]. In corticospinal fluid (CSF), approximately 0.11 ng/mL of misfolded SOD1 was found to exist; however, there was no difference in the amounts of misfolded SOD1 between ALS patient groups (96 patients) and the neurological controls (38 controls). Given that misfolded SOD1 has been immunohistochemically detected inside motor neurons, it is particularly interesting to test if their ELISA can quantitatively detect significantly more amounts of misfolded SOD1 in lysates of spinal motor neurons from non-SOD1 sALS cases than those of controls.

Regarding pathogenic conformations, if any, of SOD1 in sALS, it is notable that wild-type SOD1 oxidized with H_2_O_2_ shares conformational epitopes to C4F6 antibody with fALS-causing mutant SOD1 [51]. As mentioned above, a primary result of H_2_O_2_-oxidation on SOD1 *in vitro* is the sulfonylation at Cys^111^ [52], but it remains obscure how such oxidation affects the conformation of SOD1 proteins. Given that there has been no evidence to support the sulfonylation at Cys^111^ of SOD1 in sALS cases, it will be first necessary in a future to examine the spinal cord tissues from sALS patients by using an antibody recognizing sulfonylated Cys^111^ of SOD1.

In addition, several other types of oxidation-induced aggregation have been proposed in wild-type SOD1. For example, in a holo state, wild-type SOD1 exhibits a bicarbonate anion-dependent peroxidase activity involving carbonate anion radical, which then oxidizes a unique Trp residue in SOD1 and induces aggregation with non-disulfide covalent cross-links [56]. When Zn-deficient form of SOD1 is incubated with CuCl_2_ and ascorbic acid, furthermore, metal-catalyzed oxidation occurs mainly at metal-binding His residues in SOD1 and forms aggregates [44, 57]. SOD1 polypeptide in the disulfide-reduced apo state is also susceptible for oxidation; either H_2_O_2_ or oxidized glutathione oxidizes free thiols at Cys residues to form disulfide-linked multimers [18, 19, 58]. SOD1 is thus considered to undergo conformational changes in oxidative conditions *in vitro*; however, little evidence has been available on the presence of oxidized SOD1 in ALS patients. In that sense, a recent study by Guareschi and coworkers has provided valuable biochemical data supporting aberrant oxidation of SOD1 under pathological conditions [59].

To determine whether wild-type SOD1 is indeed oxidized in sALS cases, Guareschi and coworkers have first immunoprecipitated SOD1 from lymphoblasts derived from ALS patients and non-neurologic controls and then analyzed the presence of oxidized carbonyl groups in those immunoprecipitated SOD1 by derivatization with 2,4-dinitorophenylhydrazine (DNPH) [59]. Amounts of SOD1 with oxidized carbonyl groups, which was called iper-oxidized SOD1, were found to be significantly higher in a subset of sALS patients with bulbar onset compared with fALS with *sod1* mutations and controls. Similar to mutant SOD1 in fALS cases, furthermore, iper-oxidized wild-type SOD1 appeared to form inclusions in lymphoblasts of sALS patients with bulbar onset. While it still remains unknown in a molecular level how SOD1 is oxidized to form iper-oxidized species, this study will be particularly relevant to the extent that oxidation of wild-type SOD1 is a pathological event describing a subset of sALS cases.

## 5. Conclusions

As described above, I have briefly reviewed increasing numbers of studies that focus upon roles of wild-type SOD1 in pathogenesis of sALS cases, but it still remains quite obscure whether SOD1 is involved in a pathomechanism of the ALS cases without mutations in *sod1* gene. Nonetheless, wild-type SOD1 may somehow mediate toxicity to degenerate motor neurons; for example, astrocytes from both fALS and sALS patients are similarly toxic to motor neurons and that knockdown of *sod1* gene in those astrocytes significantly attenuates the toxicity toward motor neurons [60]. Besides, wild-type SOD1 seems to adopt abnormal conformations at least in a subset of sALS cases. In order to clarify possible roles of wild-type SOD1 in ALS pathologies, further development of new SOD1 antibodies will be promising that can recognize the conformational abnormality of SOD1 proteins. For that purpose, structural and biochemical characterization on a misfolding process of purified SOD1 proteins *in vitro* is required. Furthermore, experimental models for non-SOD1 sALS cases, which have not been established so far, will be an excellent platform for biochemical as well as immunocytochemical characterization of wild-type SOD1 and definitely facilitate the understanding of pathogenic/pathological roles of wild-type SOD1 in ALS cases.
